# Formulation Optimization of Arecoline Patches

**DOI:** 10.1155/2014/945168

**Published:** 2014-02-23

**Authors:** Pao-Chu Wu, Pi-Ju Tsai, Shin-Chen Lin, Yaw-Bin Huang

**Affiliations:** ^1^School of Pharmacy, College of Pharmacy, Kaohsiung Medical University, 100 Shih-Chuan 1st Road, Kaohsiung 80708, Taiwan; ^2^Department of Business Administration, I-Shou University, No. 1, Section 1, Syuecheng Road, Dashu, Kaohsiung 84001, Taiwan

## Abstract

The response surface methodology (RSM) including polynomial equations has been used to design an optimal patch formulation with appropriate adhesion and flux. The patch formulations were composed of different polymers, including Eudragit RS 100 (ERS), Eudragit RL 100 (ERL) and polyvinylpyrrolidone K30 (PVP), plasticizers (PEG 400), and drug. In addition, using terpenes as enhancers could increase the flux of the drug. Menthol showed the highest enhancement effect on the flux of arecoline.

## 1. Introduction

Arecoline (N-methyl-1,2,5,6-tetrahydropyridine-3-carbonic acid methyl ester) is a major alkaloid in the betel nut extract and has been used in various types of ailment treatments in ancient Arabic and Chinese cultures. Arecoline is a muscarinic cholinergic receptor agonist and has been shown to improve Alzheimer presenile dementia in certain patients after parenteral administration [1–5]. But, due to its short half-life in the blood, the transdermal delivery dosage form could avoid the absorption variability. Furthermore, transdermal patches offer advantages such as bypass hepatic first pass effect, maintain a constant and prolonged drug level, decreased frequency of dosing, and easy termination of medication leading to patient compliance [6]. Hence, the arecoline transdermal patches were developed in this study.

There are three main types of transdermal patches systems: (1) adhesive systems: the drug disperse or dissolve in adhesive, (2) matrix type systems: the drug in a matrix polymer, and (3) reservoir systems [7]. The adhesive system was simple and chosen to prepare arecoline-loaded transdermal patch in this study. Eudragit polymers have high capacity for drugs loading and are well tolerated by skin, hence Eudragit RS 100 (ERS) and Eudragit RL 100 (ERL) were used as adhesive polymers [8]. Polyvinylpyrrolidone K30 (PVP) was used to increase the adhesive of Eudragit polymers [9]. In general, polymers used in pharmaceutical formulations are brittle and require the addition of a plasticizer to ease the thermal workability, improving the mechanical properties and modifying the drug release from polymeric systems [10–13]. The polyethylene glycols 400 (PEG) was used as plasticizer. In this study, thin adhesive arecoline film patches composed of three types of copolymers (ERS, ERL, and PVP), plasticizer (PEG), and drug were designed. Furthermore, the terpenes were used as enhancer to increase the flux of drug from the patches.

In the development of patch formulations, an important thesis was to develop an applicable formulation with ample penetration rate (flux) and adhesion to skin in a short time period with minimum experimental trials. To reach the target, response surface methodology (RSM) including polynomial equations has been widely used [14–19]. The optimization procedure included two steps: a systematic formulations plan to minimize the number of trials, and the response surfaces analysis to realize the effect of causal factors and to obtain the optimal formulations with target goals. A computer optimization technique based on an RSM was used to evaluate the effects of components of formulations on patch adhesion and drug penetration rate (flux) through rat skin and to obtain applicable formulations.

## 2. Materials and Methods

### 2.1. Materials

The following reagents were used: arecoline hydrobromide (Sigma-Aldrich, St. Louis, Missouri, USA), Eudragit RL 100 (ERL), Eudragit RS 100 (ERS) (Rohm Gmbh, Sontheim/Brenz, Germany), polyvinylpyrrolidone K 30 (PVP), limonene, cineole, carvone, 1-octasulponic acid, and menthol (Tokyo Chemical Industry, Tokyo, Japan), polyethylene glycols 400 (PEG) (Merck Chemicals, Darmstadt, Germany). The free base of arecoline was prepared and purified by repeated extraction of aqueous arecoline hydrobromide solution (adjusted to pH 10 by 2 M NaOH) with petroleum ether [20]. All other chemicals and solvents were of analytical reagent grade.

### 2.2. Stability Measurement

The stability of arecoline in phosphate-citric acid buffer of pH 5.5 and pH 7.4 with different levels of ethanol was assessed. Arecoline was dissolved in phosphate buffer solution and stored in an ampule at 37°C, 75% RH. At the designated time, five hundred milliliters of sample was withdrawn from the ampule and stored at −20°C until analyzed by HPLC [1].

### 2.3. Preparation of Arecoline Patches

For systemic evaluation of the influence of each component of formulations on the desired goals such as penetration rate and adhesion of patch, the modified crossed mixture-process factors design [21] was applied to prepare various systematic model formulations. There were three restricted mixture components (ERL = *X*_1_, ERS = *X*_2_, PVP = *X*_3_; 0.2 ≤ *X*_1_, *X*_2_, and *X*_3_ ≤ 0.6; *X*_1_ + *X*_2_ + *X*_3_ = 1) and a full 2^2^ factorial structure for the level of plasticizer (PEG 400 = *X*_4_; 0.2 ≤ *X*_4_ ≤ 0.33) and drug (arecoline = *X*_5_; 0.05 ≤ *X*_5_ ≤ 0.2). The range of each process variable was set according to our preliminary screen experiments (data not shown). The statistical software Design-Expert was used to generate the system formulations. The design formulations and their response data are shown in Table 1.

Patch systems were fabricated by the solvent evaporation technique [22]. The defined weight of copolymers (ERL, ERS, and PVP), plasticizer (PEG), arecoline, and/or penetration enhancer (carvone, cineole, limonene, and menthol) was dissolved in dichloromethane/isopropyl alcohol (60 : 40) mixed solvent for each formulation. The solution was poured into a glass ring of 8 cm × 8 cm placed on a glass plate covering an aluminum foil as backing film. The solvent was allowed to evaporate at ambient conditions in a hood. A final thin film of 250–300 *μ*m thickness was produced after drying for 24 h. The polymer matrix was found to be self-sticking due to the presence of copolymers along with plasticizer. The release liner was then applied to the top release surface of the thin film. Patches of 1.5 × 1.5 cm^2^ were cut and stored in individually heat-sealed aluminum-coated polyethylene foil (Tricon Chemical Corporation, Forestville, MD, USA).

### 2.4. Peel Adhesion 180° Test

The arecoline-loaded patches were cut into strips 2.5 cm wide. The samples were applied to an adherent teflon plate, smoothed with a 4.5 pound roller, and pulled from the substrate at a 180° angle at a rate of 300 mm/min [23–25]. The matrix had to strip cleanly from the plate, leaving no visually noticeable residue. The force was expressed in centiNewtons per centimeter (cN/cm) width of adhesive tape under test. Peel adhesion values represented the mean of three samples.

### 2.5. *In Vitro* Skin Permeation Studies

The cumulative amount and flux of arecoline from patch formulations through excised rat skin were determined using a modified glass diffusion cell. The stratum corneum side of the rat skin was kept in intimate contact with the release surface of the patch under test placed between the two halves of the diffusion cell. The effective diffusion area was 3.46 cm^2^. The receptor compartment contained 20 mL of pH 5.5 phosphate buffer maintained at 37 ± 0.5°C by thermostatic water pump during the experiment. At determined intervals, the receptor medium of 0.5 mL was withdrawn. In the meanwhile, an equal volume of fresh receptor medium was replaced immediately to maintain a constant volume. This dilution of the receiver content was taken into account when evaluating the penetration data. The drug level of the sample was then analyzed by a HPLC method described in an earlier study [1]. A Merck Lichrocart C18 column (55 × 4 mm I.D., particle size 5 *μ*m) (Merck Chemicals, Darmstadt, Germany) was used. The mixture solution of 15 mM Phosfate buffer containing 3.75 mM 1-octasulphate acid sodium (adjusted to pH 3.0 by phosphoric acid) and methanol at the ratio of 67 : 33 was used as mobile phase. The flow rate was at 1 mL/min. The detection wavelength was set at 210 nm. Each permeation data point expressed the average of three determinations.

### 2.6. Statistical Analysis of Data

The cumulative amount of the arecoline at determined intervals was plotted as a function of time. The flux of arecoline was calculated by the linear regression analysis.

The flux, peel adhesion, and formulation variables of all model formulations were treated by Design-Expert software. The statistical analysis process included stepwise linear regression and response surface analysis. The statistical parameters including the multiple correlation coefficient (*r* ^2^), adjusted multiple correlation coefficient (adjusted *r* ^2^), coefficient of variation (C.V.), and lack-of-fit proven by Design-Expert software [16] were used to evaluate and select the best-fitting mathematical model.

## 3. Results and Discussion

### 3.1. Stability

According to a previous study [20], it was reported that arecoline hydrobromide and free base of arecoline are extremely sensitive to its vehicle environment, especially in aqueous solutions with a pH close to the drug pKa of 6.84. Hence, the stability of arecoline in pH 5.5 and pH 7.4 phosphate buffers containing 0~75% ethanol were evaluated. The result showed that after 72 h of incubation, the residual percentages of drugs were 79.4 and 98.9% for pH 7.4 and pH 5.5 phosphate buffer, respectively, showing that arecoline was more stable in pH 5.5 phosphate buffer. The stability of drug in pH 7.4 phosphate buffer increased with that increase in ethanol concentration (data not shown). But the phenomenon was not observed in pH 5.5 phosphate buffer. The pH value of normal skin is close to pH 5.5; hence, the pH 5.5 phosphate buffer was used as receptor medium in the *in vitro* permeation study to ensure the chemical stability of arecoline during experimental.

### 3.2. Arecoline-Loaded Patches Preparation

**Figure 1:**
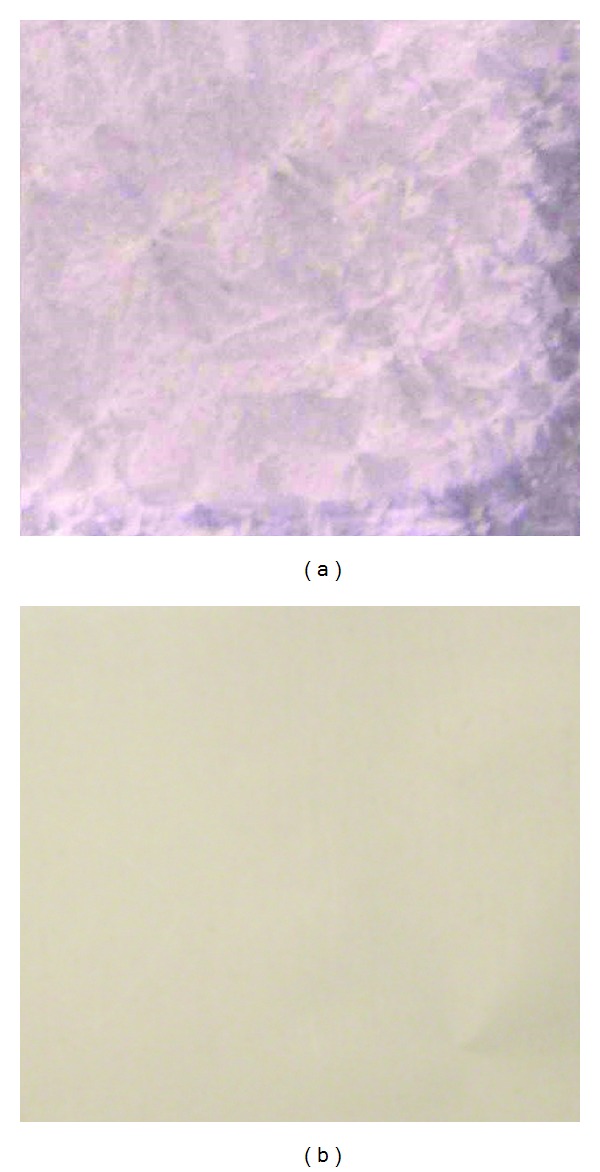
The appearance of drug-loaded patch prepared from arecoline hydrobromide (a) and arecoline base (b).

The free base and hydrobromide of arecoline were used to prepare the drug-loaded patches. As shown in Figure 1, the surface of the arecoline hydrobromide-loaded patch was coarse and some precipitate was observed, whereas, the surface of free base arecoline-loaded patch was comparable smooth which indicated that free base arecoline was easier to disperse in ERL/ERS/PVP copolymers. Furthermore, the flux of arecoline base through rat skin was higher than that of arecoline hydrobromide (data not shown). Hence, the free base of arecoline was used to prepare the drug-loaded patches in this study.

### 3.3. Formulation Optimization

**Figure 2:**
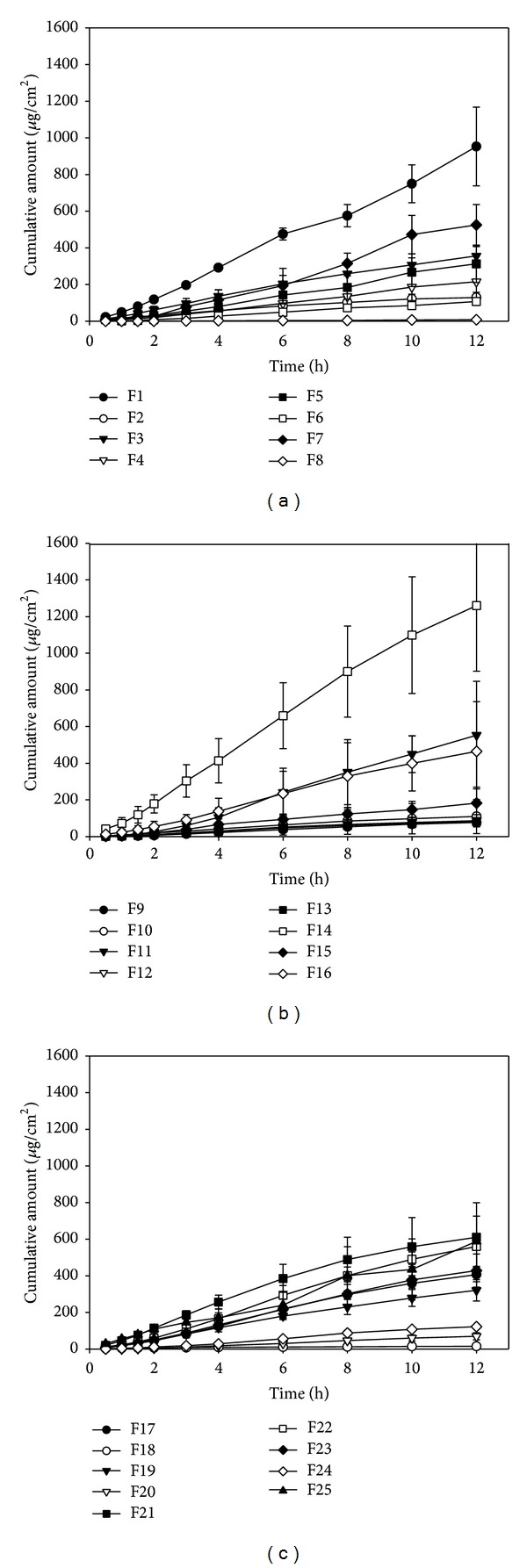
*In vitro* penetration-time profile of model arecoline-loaded patch formulations through rat skin (*n* = 3).

In the exploitation of pharmaceutical products, an important subject is to obtain an applicable formulation with desirable goals in a short time period with minimum trials. The statistical method, RSM, has successfully been used in this region of development of pharmaceutical formulations [17, 19]. For patch formulations, higher flux through skin to maintain therapeutic drug levels in the blood and an appropriate adhesion of patch for adhering to the skin were the two most important goals. It has been demonstrated that when the patch fails to adhere, the effectiveness of product should decrease [26]. In general, increasing the adhesion of patch should decrease the fluidity of the drug in the formulation, thereby resulting in the decrease of flux. Hence, both properties of flux and adhesion of each patch must be jointly considered in layout of an applicable patch formulation. The RSM with “crossed” design was used in this study to evaluate the effects of formulation variables including level of ERL (*X*_1_), ERS (*X*_2_), and PVP (*X*_3_), PEG (*X*_4_), and arecoline (*X*_5_) on the flux of drug and adhesion of formulations.  Figure 2 shows the permeation profiles of these model arecoline patches through excised rat skin. The permeation profiles of arecoline exhibited a zero-order permeation at a constant flux (*r* ^2^ > 0.9637). The fluxes of all model formulations were calculated and listed in Table 1. The adhesion of patches was also determined. It can be seen that the responses of these model formulations have significant differences: flux is from 0.96 to 111.11 *μ*g/cm^2^/h and adhesion is from 5.00 to 254.63 cN/cm. The wide variation demonstrated that both properties of formulations were remarkably influenced by the composition of the patches.

To evaluate the quantitative effects of the different combination proportions of these formulation variables on the flux and adhesion, the response surface models were calculated with Design-Expert software. The model describing the flux can be written as

(1) Flux=+43.73−68.6X4+65.4X5−59.5X42+112.7X2X4−96.2X2X5−45.7X3X5+39.0X4X5+139.2X2X42−92.6X2X4X5+11.90X3X4X5.

**Figure 3:**
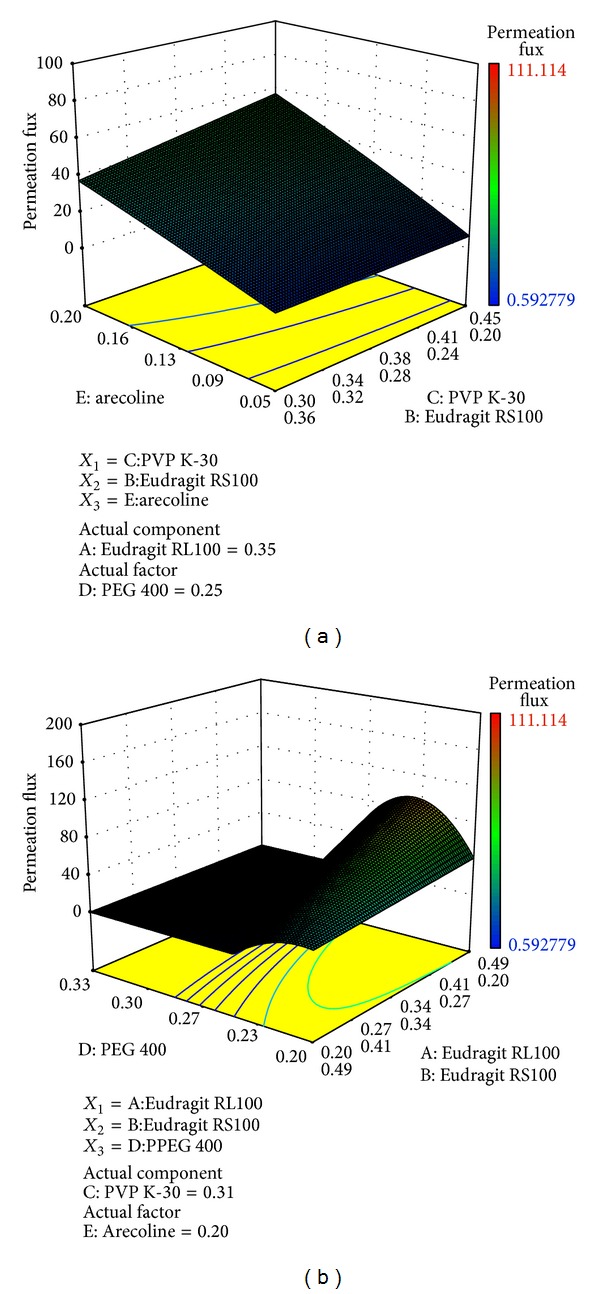
The three-dimensional diagrams illustrating the effect of the level of PEG and arecoline on the permeation capacity of drug from patch.

The significance probability value (*P* value), lack of fit, *r*-square, adjusted *r*-square, and C.V. were 0.002, 0.4334, 0.9454, 0.8361, and 38.05 for flux model and <0.0001, 0.0744, 0.8516, 0.7725, and 49.07, respectively, showing that the assumed mathematic model was significant and valid for the considered response. The values of the coefficients in the mathematic equation were associated with the effect of these formulation variables on the response (flux). A positive term presents a synergistic effect, while a negative sign reveals an antagonistic effect on the response [17, 27]. From the mathematic equation of flux, it showed that PEG (*X*_4_) and arecoline (*X*_5_) had the greatest potential influence on the response. As shown in Figure 3(a), the flux increased with increase in drug loading. The patch containing appropriate amount of PEG showed highest flux (Figure 3(b)). This was in good agreement with previous studies, which reported that the plasticizers such as glycerin, polyethylene glycol, and sorbitol can change release rate of the active pharmaceutical ingredients contained in the formulations of transdermal drug delivery systems. Release rate of the drug can be adjusted by changing the type and concentration of the plasticizer [11, 12]. The mechanism of plasticizers might be due to reduced polymer-polymer chain secondary bonding, forming secondary bonds with the polymer chains instead, and then improving the properties and appearance of the forming film and control of the release rate of the therapeutic compounds [28].

The mathematic equation (2) describing the adhesion can be written as

(2) Adhesion=+8.5X1+33.3X2+254.9X3−11.4X1X4+11.9X1X5−5.1X2X4+7.20X2X5+181.3X3X4+8.3X3X5.

In the case of the adhesion model, it showed that the most significantly influencing variables on adhesion of the patch were *X*_3_ (PVP) and interaction of *X*_3_ *X*_4_ (PVP and PEG). This might be attributed to PVP being an adhesive copolymer and Eudragit RS and Eudragit RL being nonadhesive copolymers [24–26]. And the adhesion of the patch would be increased by plasticizer (PEG) incorporated [24].

According to previous study of the pharmacokinetics and pharmacodynamics of intravenously administered arecoline in subjects with Alzheimer's disease [1], at the dose that optimized memory, the mean plasma level was 0.31 ± 0.14 ng/mL and the clearance was 13.6 ± 5.8 L/min. The mean plasma level represents a target average arecoline concentration that would maintain a desired pharmacological effect. Assuming that, at steady state, the elimination rate equals the input rate after a transdermal patch administration, the required flux of a patch dosage form to produce such a concentration can be calculated from the following equation: required flux = concentration × Cl = 253 *μ*g/h, assuming that a reasonable size for the topical application area is 25 cm^2^ (5 cm × 5 cm). In general, drug penetration capability through rat skin is 3–5 times higher than through human skin. Therefore, the desired flux of the optimal patch was set at above 50 *μ*g/cm^2^/h in this study. The adhesion was set at a range of 30–60 cN/cm, because the patch could be removed without residue remaining attached to the Teflon plate when its adhesion was above 30 cN/cm. The optimal formulation with flux of 57.42 *μ*g/cm^2^/h and adhesion of 35.63 cN/cm, composed of *X*_1_ = 0.2, *X*_2_ = 0.6, *X*_3_ = 02, *X*_4_ = 0.33, *X*_5_ = 0.26, was obtained from the RSM.

**Figure 4:**
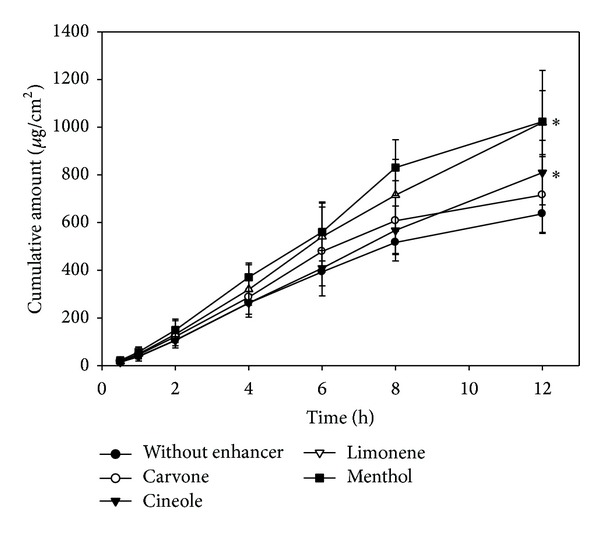
*In vitro* penetration-time profile of arecoline-loaded patch with 5% terpenes as enhancers through rat skin (*n* = 3) (*significant difference *P* < 0.05).

Terpenes are a series of natural compounds which are comprised of isoprene (C5H8) units and have been reported to reveal an enhancement effect on permeation rate of hydrophobic and hydrophilic drugs such as alfuzosin, bufalin, meloxicam, propranolol, and tamoxifen [16, 29–33]. Hence, different terpenes including cineole, carvone, limonene, and menthol were used in this study. In order to enhance the flux and not affect the adhesion of patch, 5% enhancers were incorporated. As expected, the cumulative amounts of arecoline permeation through the skin were significantly increased by incorporated enhancers (Figure 4). The rank order of increased effect for drug penetration rate was menthol > limonene > cineole > carvone. It was found that terpenes with a hydroxyl group such as menthol were most effective on the transport of arecoline. This result was coincidental to previous studies [30], which reported that penetration enhancers had functional groups with hydrogen-bonding ability effectively improving the drug transport through skin. The enhancement mechanisms of menthol might be attributed to increase the arecoline partition into the SC, lipid extraction, and perturbation of the macroscopic barrier properties of the skin [30, 33]. However, arecoline patch with 5% menthol shows the highest flux of 92.56 ± 15.59 *μ*g/cm^2^/h. The result demonstrated that the desired pharmacological effect could be obtained by the administration of a reasonable size (<15 cm^2^) of experimental arecoline-loaded patch.

## 4. Conclusion

An optimal arecoline-loaded patch exhibited good adhesion and appropriate flux was obtained by using response surface methodology. The arecoline-loaded patch with 5% menthol shows the highest flux of 92.56 ± 15.59 *μ*g/cm^2^/h. The desired pharmacological effect could be obtained by administration of a reasonable size of arecoline-loaded patch.

## Figures and Tables

**Table 1:** The composition and responses (penetration rate and adhesion) of model arecoline-loaded patches.

	*X*_1_	*X*_2_	*X*_3_	*X*_4_	*X*_5_	Response	Response
	(g)	(g)	(g)	(g)	(g)	Flux (*μ*g/cm^2^/h)	Adhesion (cN/cm)
F1	0.60	0.20	0.20	0.20	0.20	80.83 ± 15.78	18.07 ± 3.94
F2	0.40	0.20	0.40	0.20	0.05	19.05 ± 3.62	49.58 ± 8.02
F3	0.20	0.20	0.60	0.25	0.20	22.24 ± 2.76	254.63 ± 37.92
F4	0.20	0.40	0.40	0.20	0.05	25.18 ± 8.29	52.83 ± 7.40
F5	0.60	0.20	0.20	0.20	0.05	26.99 ± 12.29	6.20 ± 1.08
F6	0.20	0.60	0.20	0.25	0.05	9.67 ± 4.30	6.52 ± 3.15
F7	0.33	0.33	0.33	0.23	0.13	47.14 ± 11.62	62.05 ± 7.02
F8	0.20	0.40	0.40	0.25	0.05	0.59 ± 0.09	86.55 ± 16.50
F9	0.20	0.20	0.60	0.20	0.05	7.22 ± 0.97	85.48 ± 12.15
F10	0.40	0.40	0.20	0.20	0.05	14.17 ± 0.42	40.37 ± 7.81
F11	0.20	0.60	0.20	0.33	0.20	34.60 ± 16.41	57.42 ± 15.38
F12	0.40	0.20	0.40	0.20	0.20	40.20 ± 26.53	216.38 ± 25.08
F13	0.40	0.40	0.20	0.25	0.05	6.53 ± 1.33	33.72 ± 5.85
F14	0.60	0.20	0.20	0.25	0.20	111.11 ± 42.84	33.12 ± 6.49
F15	0.20	0.40	0.40	0.20	0.05	15.86 ± 6.02	56.88 ± 4.15
F16	0.60	0.20	0.20	0.33	0.20	20.31 ± 8.12	41.12 ± 5.23
F17	0.20	0.20	0.60	0.25	0.05	11.40 ± 1.12	231.80 ± 34.61
F18	0.60	0.20	0.20	0.24	0.09	37.20 ± 4.73	22.79 ± 3.94
F19	0.40	0.20	0.40	0.25	0.05	0.96 ± 0.05	29.44 ± 4.31
F20	0.20	0.40	0.40	0.20	0.20	36.85 ± 5.44	163.69 ± 24.97
F21	0.20	0.60	0.20	0.20	0.05	7.06 ± 0.62	5.00 ± 0.88
F22	0.20	0.20	0.60	0.33	0.20	52.14 ± 11.23	48.39 ± 7.23
F23	0.20	0.60	0.20	0.20	0.20	54.48 ± 19.79	59.97 ± 13.78
F24	0.20	0.20	0.60	0.21	0.16	38.66 ± 0.23	76.93 ± 12.29
F25	0.40	0.40	0.20	0.25	0.20	43.12 ± 15.53	22.98 ± 2.51

(1) The amount of each formulation was given an area of 64 cm^2^ of arecoline-loaded patch.

(2) The *X*_1_, *X*_2_, *X*_3_, *X*_4_, and *X*_5_ were the weight of Eudragit RL 100, Eudragit RS 100, PVP K30, PEG 400, and arecoline.
